# Conserved role for PCBP1 in altered RNA splicing in the hippocampus after chronic alcohol exposure

**DOI:** 10.1038/s41380-023-02184-y

**Published:** 2023-08-03

**Authors:** Luana Carvalho, Hu Chen, Mark Maienschein-Cline, Elizabeth J. Glover, Subhash C. Pandey, Amy W. Lasek

**Affiliations:** 1 Center for Alcohol Research in Epigenetics, Department of Psychiatry, University of Illinois at Chicago, Chicago, IL 60612 USA; 2 Research Informatics Core, University of Illinois at Chicago, Chicago, IL 60612 USA; 3 Jesse Brown VA Medical Center, Chicago, IL 60612 USA; 4 Present Address: Department of Pharmacology and Toxicology, Virginia Commonwealth University, Richmond, VA 23298 USA

**Keywords:** Molecular biology, Neuroscience, Genetics

## Abstract

We previously discovered using transcriptomics that rats undergoing withdrawal after chronic ethanol exposure had increased expression of several genes encoding RNA splicing factors in the hippocampus. Here, we examined RNA splicing in the rat hippocampus during withdrawal from chronic ethanol exposure and in postmortem hippocampus of human subjects diagnosed with alcohol use disorder (AUD). We found that expression of the gene encoding the splicing factor, poly r(C) binding protein 1 (*PCBP1*), was elevated in the hippocampus of rats during withdrawal after chronic ethanol exposure and AUD subjects. We next analyzed the rat RNA-Seq data for differentially expressed (DE) exon junctions. One gene, *Hapln2*, had increased usage of a novel 3′ splice site in exon 4 during withdrawal. This splice site was conserved in human *HAPLN2* and was used more frequently in the hippocampus of AUD compared to control subjects. To establish a functional role for PCBP1 in *HAPLN2* splicing, we performed RNA immunoprecipitation (RIP) with a PCBP1 antibody in rat and human hippocampus, which showed enriched PCBP1 association near the *HAPLN2* exon 4 3′ splice site in the hippocampus of rats during ethanol withdrawal and AUD subjects. Our results indicate a conserved role for the splicing factor PCBP1 in aberrant splicing of *HAPLN2* after chronic ethanol exposure. As the *HAPLN2* gene encodes an extracellular matrix protein involved in nerve conduction velocity, use of this alternative splice site is predicted to result in loss of protein function that could negatively impact hippocampal function in AUD.

## Introduction

Disease-related changes in the cellular machinery that processes RNA to its mature form can lead to mis-splicing of RNA and the subsequent production of aberrant protein products and compromised cellular function [1]. Indeed, altered RNA splicing contributes to neurological and psychiatric disorders such as Alzheimer’s disease and major depressive disorder [2–4], as well as substance use disorders [5, 6]. Studies in several species have reported that alcohol exposure results in altered RNA splicing. For example, in fruit flies, changes in the expression of specific transcripts in the mushroom body are associated with odor cue-associated ethanol memory, and knockdown of genes encoding splicing factors reduces this type of memory [7]. Chronic ethanol exposure induces RNA splicing changes in the cortex of monkeys and rodents [8, 9] and acute ethanol exposure leads to differential expression of exons in the synaptic transcriptome in mouse hippocampus [10]. In humans, a recent RNA-Seq study reported genome-wide changes in splicing in the superior frontal cortex, nucleus accumbens, basolateral amygdala, and central nucleus of amygdala of individuals diagnosed with alcohol use disorder (AUD) [6]. Although changes in the expression of heat shock protein family A member 6, and a variety of long noncoding RNAs involved in RNA splicing were observed, the exact mechanisms underlying RNA splicing changes in mammals after chronic ethanol exposure have not been explored.

We have been investigating molecular alterations in the rat hippocampus during withdrawal after chronic ethanol exposure and in postmortem human hippocampus of AUD subjects. We recently published an RNA-Seq study of the rat hippocampus after chronic alcohol exposure and its withdrawal [11], in which we discovered that withdrawal from chronic ethanol exposure resulted in increased expression of genes involved in RNA splicing in the hippocampus. Here, we extended these findings by validating the expression changes in genes encoding splicing factors in the hippocampus of male and female rats during withdrawal from chronic ethanol exposure and AUD subjects. Moreover, we analyzed differentially expressed (DE) junctions from male rat RNA-Seq data that could be related to the increased expression of splicing factors during withdrawal after chronic ethanol exposure. We next focused on one of these splicing factors, poly r(C) binding protein 1 (PCBP1), a multifunctional RNA binding protein that acts as a splicing factor by binding to intronic cytosine-rich sequences to control exon inclusion or exclusion [12–14]. In addition to regulating RNA splicing, PCBP1 also regulates nuclear export, stabilization, localization, transcription, and translation of its RNA targets [15–17].

Some of the known RNA targets of PCBP1 encode proteins that are involved in neuroinflammatory processes, inherited peripheral neuropathies [18] and axonal outgrowth [19, 20]. Here, we discovered that PCBP1 associates with a splice site region in the pre-mRNA of one of the genes with DE junctions, hyaluronan and proteoglycan link protein 2 (*HAPLN2*). We also demonstrate that increased PCBP1 association with *HAPLN2* pre-mRNA is conserved in human hippocampus, implicating this splicing factor in *HAPLN2* alternative splicing. Together our results indicate that withdrawal from chronic alcohol intake induces changes in RNA isoforms in the hippocampus, alters the expression of splicing factors, and highlights PCBP1 as an important regulator of alternative splicing in AUD.

## Materials and methods

### Chronic ethanol treatment in rats

Adult male and female Sprague Dawley rats (75–85 days old) were purchased from Envigo (Indianapolis, IN, USA) and individually housed in a temperature and humidity-controlled room with a 12-h light/dark cycle, with food and water provided *ad libitum* prior to beginning the chronic ethanol treatment protocol, as reported by us earlier [21]. When female and male rats reached 250 g and 350 g body weight respectively, they were pair-fed with the nutritionally complete Lieber-DeCarli control or ethanol liquid diet (Bio-Serv; Frenchtown, NJ, USA) as their only source of food and fluid. All rats were first fed with control diet for 3 days and then were assigned to different treatment groups based on their weights to assure no discrepant difference between groups. The control group continued with the control diet for 16 days, while the ethanol group was gradually introduced to ethanol over 7 days (increasing from 1.8 to 8.1%). Rats were then maintained on a 9% ethanol diet for 15 days. Ethanol-withdrawn rats were switched to control liquid diet for 24 h after removal of the ethanol liquid diet whereas the ethanol group continued on ethanol diet for one additional day. Previous use of this model of ethanol exposure has demonstrated that blood alcohol levels are zero at 24 h withdrawal [21–23]. Rats were pair fed and their liquid diet intake and body weights were closely monitored. There was no significant difference in body weights between the control and ethanol liquid diet fed groups or change in body weights during the experiment similar to our previous studies [21, 24]. At the end of the experiment rats were anesthetized, rapidly decapitated and the dorsal hippocampus was dissected, frozen on dry ice, and stored at −80 ^o^C. Animal care was conducted according to the National Institutes of Health *Guide for the Care and Use of Laboratory Animals* and all experimental procedures were approved by the University of Illinois at Chicago Animal Care Committee. This study was conducted in a nonblinded fashion.

### Human subjects

Human postmortem hippocampus tissue from 25 subjects (*n* = 15 males and 10 females) diagnosed with AUD and 24 control subjects (*n* = 17 males and 7 females) was obtained from the New South Wales Brain Tissue Resource Centre (University of Sydney, Australia). Individuals were diagnosed with AUD according to DSM-IV criteria. Demographic characteristics of subjects are shown in Table 1. RNA was isolated and analyzed by qPCR and tissue was also used for RNA immunoprecipitation (RIP). Statistical outliers were identified using the ROUT method [25] and removed from statistical analysis and graphs. For *PCBP1* expression analysis one AUD and one control subject were removed. For RIP analysis 2 samples (one male and one female AUD subject) were removed.

**Table 1:** Demographic characteristics of control and alcohol use disorder (AUD) subjects.

Characteristics	Control (*n* = 24)	AUD (*n* = 25)	*P* value
Sex	F (7) - M (17)	F (10) - M (15)	**-**
Age, years	58.33 ± 2.05	56.96 ± 2.00	*p* = 0.63
PMI (h)	34.00 ± 2.78	37.98 ± 3.25	*p* = 0.358
Brain pH	6.62 ± 0.06	6.57 ± 0.05	*p* = 0.497
Total drinking years	29.14 ± 3.48	31.90 ± 2.03	*p* = 0.484
Ethanol daily use (g)	11.42 ± 3.53	166.76 ± 24.65	*p* < 0.0001
Drinks per week	6.62 ± 2.46	86.52 ± 15.39	*p* < 0.0001
Cigarette pack years	22.23 ± 7.38	34.25 ± 0.12	*p* = 0.124
Cause of death (*n*)			**-**
Cancer	1	-	
Cardiac	21	12	
Cardiovascular	1	-	
Hepatic	-	3	
Infection	-	2	
Respiratory	2	4	
Stroke	-	1	
Toxicity	4	6	
Vascular	1	-	
Unknown	-	2	

### RNA-Seq and bioinformatic analysis of differentially expressed junctions

RNA-Seq was performed previously on dorsal hippocampi from six male rats per group and is described in Chen et al. [11]. No RNA-Seq was performed on females. Males RNA-Seq data is available in the Gene Expression Omnibus (GEO) database, accession number GSE171051. DE junctions were identified after aligning the sequences to the NCBI Rnor 6.0 genome using STAR (Spliced Transcripts Alignment to a Reference), a splice-aware aligner that allows the unbiased de novo detection of canonical junctions as well as noncanonical splices [26]. Differential statistics of junction counts were computed using exactTest() in edgeR [27] to compare between each pair of groups and normalizing abundances with TMM. *P* values were corrected for multiple testing using the Benjamini–Hochberg FDR correction [28] with a cutoff of *q* < 0.05 to define statistical significance.

### Polymerase chain reaction (PCR)

Real-time quantitative PCR (qPCR) was performed on RNA from dorsal hippocampi from ten male and six female rats per group. For postmortem hippocampus tissue, qPCR was performed in 24 controls (17M/7F) and 25 AUD subjects (15 M/10 F) (demographic details are in Table 1). Total RNA was isolated from frozen tissue using Qiazol reagent (Qiagen) and further purified using the miRNeasy Mini kit (Qiagen). After treatment with RNase-Free DNase (Qiagen), RNA was converted to cDNA using the High-Capacity cDNA Reverse Transcription Kit (Applied Biosystems). SYBR Green Supermix (Bio- Rad) was used for qPCR. Relative mRNA levels were determined using the 2^−ΔΔCt^ method with *Hprt* and *Gusb* as reference genes for rats, and *ACTB* and *B2M* for humans. Primers used for splicing factor validation are listed in Supplementary Table 1 for rats and Supplementary Table 2 for humans.

To quantify percent spliced isoforms (PSI) of *Hapln2*, conventional PCR was performed on RNA from dorsal hippocampi of control (*n* = 10) and ethanol withdrawn (*n* = 10) male rats and on RNA from postmortem human hippocampus (*n* = 24 control, 17 M and 7 F, and *n* = 25 AUD, 15 M and 10 F). Total RNA was isolated, treated with DNase and used for cDNA synthesis. PCR was performed using a forward primer located in exon 3 and a reverse primer located in exon 4. Primers for both rats and humans are listed in Supplementary Table 3. PCR products were analyzed on a 1% agarose gel, bands were visualized using SYBR™ Safe DNA Gel Stain (Invitrogen) and purified using the QIAquick Gel Extraction Kit (Qiagen). Purified DNA (10 ng/μl) was sent to the Genomics Research Core at UIC for Sanger Sequencing. Image J was used to calculate the relative abundance of each isoform on the gel image as PSI, which was calculated as intensity of a given isoform divided by the intensity of the sum of all isoforms, multiplied by 100.

### RNA Immunoprecipitation (RIP)

RIP was performed on dorsal hippocampi from a separate cohort of 20 male rats (*n* = 9 control and *n* = 11 ethanol-withdrawn) and 12 female rats (*n* = 6 control and *n* = 6 ethanol-withdrawn) and on postmortem human hippocampus tissue from 24 (17 M and 7 F) control and 25 (15 M and 10 F) subjects diagnosed with AUD. Frozen tissue was homogenized on ice in phosphate buffered saline (PBS) containing proteinase inhibitor cocktail (PIC) (Thermo Fisher Scientific) and fixed in 16% methanol-free formaldehyde for 10 min at 23 °C. Homogenate was lysed with nuclear lysis buffer (1.3% Sucrose, 40 mM Tris-HCl, 20 mM MgCl2, 4% Triton X-100) containing Protein Inhibitor Cocktail and RNase inhibitor (Thermo Fisher Scientific). The post nuclear fraction was collected by spinning the cells at 2500 x g for 15 min at 4 °C and resuspended in RIP buffer (150 mM KCl, 25 mM Tris-HCl, 5 mM EDTA, 0.5 mM DTT, 0.50% NP 40) containing PIC and RNase inhibitor. Subsequently, 180 μl of post nuclear fraction was sonicated (Covaris, Woburn, MA, USA) to shear RNA to ~200 bp fragments then pelleted by centrifugation at 13,000 × *g* for 10 min at 4 °C. 140 μl of clarified supernatant was incubated overnight at 4 °C with 5 μg of mouse monoclonal anti-PCBP1 antibody (AB_2299322, #sc137249, Santa Cruz Biotechnology), and the remaining 40 μl was used as input. PCBP1 antibody has been validated in rat tissue and used for western blot and immunoprecipitation [14, 29, 30]. Positive and negative controls used anti-Hur and anti-IgG antibodies respectively (#03-102 Millipore Sigma). Magnetic Protein A Dynabeads (Thermo Fisher Scientific) were added to samples and rotated for 1 h at 4 °C. Beads were washed 3 times with RIP buffer and once with PBS, then samples were treated with DNase (Qiagen) and Proteinase K (Thermo Fisher Scientific). RNA extraction was done in both pulldown and input samples by the addition of Qiazol reagent (Qiagen) and then RNA was purified with the miRNAeasy Mini Kit (Qiagen). RNA was reverse transcribed using the High-Capacity cDNA Reverse Transcription Kit (Applied Biosystems) following manufacturer’s instructions and analyzed using qPCR with primers specific for *Hapln2* (Supplementary Table 3). Relative enrichment of PCBP1 binding was calculated by normalizing to input using the 2^−ΔΔCT^ method [31].

### Statistical analysis

**Fig. 1:**
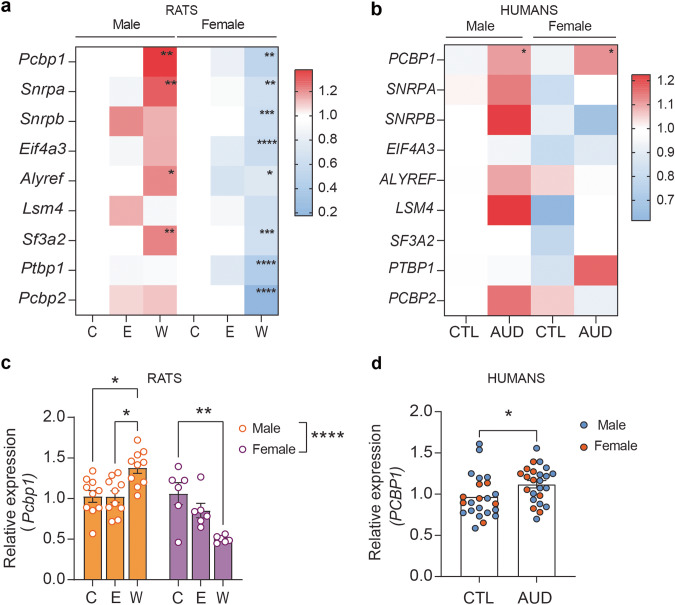
Altered expression of genes encoding splicing factors in the hippocampus of ethanol withdrawn rats after chronic exposure and human subjects with AUD. **a, c** Rats were fed control liquid diet (C), ethanol liquid diet (E), or ethanol liquid diet followed by 24 h of withdrawal from ethanol diet (W). The dorsal hippocampus was dissected and RNA isolated and subjected to qPCR (*n* = 10 males and 6 females per group). **b, d** RNA was isolated from the hippocampus of control (CTL, *n* = 17 male and 7 females) human subjects and those with AUD (*n* = 15 male and 10 female) and subjected to qPCR. **a** Heat map showing the expression of *Pcbp1, Snrpa, Snrpb, Eif4a3, Alyref, Lsm4, Sf3a2, Ptbp1*, and *Pcbp2* relative to the control group for each sex in male and female rat hippocampus. *p < 0.05, **p < 0.01, and ****p < 0.0001 when comparing withdrawal to control, by Sidak’s test after two-way ANOVA. Full statistics are reported in Supplementary Table 4. **b** Heat map showing the relative expression of *PCBP1*, *SNRPA, SNRPB, EIF4A3, ALYREF, LSM4, SF3A2, PTBP1*, and *PCBP2* in human postmortem hippocampus. Two-way ANOVA indicated a significant diagnosis effect for *PCBP1* and a significant sex effect for *SNRPA*, *SNRPB* and *LSM4*. *p < 0.05, main effect of diagnosis. **c** *Pcbp1* expression in male and female rat hippocampus, shown as mean ± SEM with individual data points as circles. *p < 0.05 when comparing withdrawal vs. control and vs. ethanol within males, **p < 0.01 when comparing withdrawal vs. control within females. ****p < 0.0001, main effect of sex. **d** *PCBP1* expression in postmortem hi*p*pocampus of male and female control and AUD subjects. *p < 0.05 Significant diagnosis effect for *PCBP1* expression. Data shown as the mean ± SEM with individual data points as circles.

**Fig. 2:**
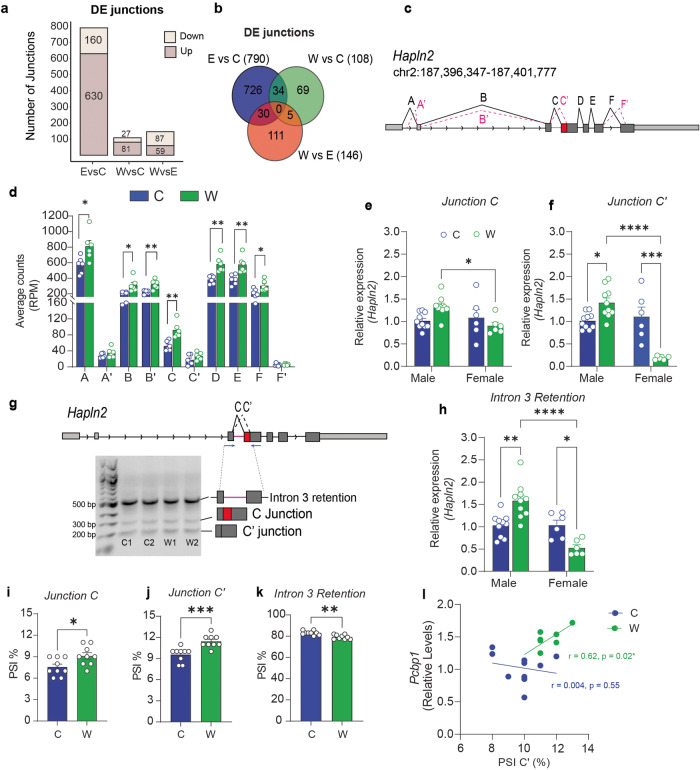
Differential junction expression in the hippocampus of chronic ethanol exposed and withdrawn rats. Male rats were fed control liquid diet (C), ethanol liquid diet (E), or ethanol liquid diet followed by 24 h of withdrawal from ethanol diet (W). The dorsal hippocampus was dissected and RNA isolated and subjected to RNA sequencing (RNA-Seq, *n* = 6 per group). Differentially expressed (DE) splice junctions were identified by using STAR aligner. **a** Stacked bar plot depicting the upregulated, downregulated, and total number of DE junctions at FDR < 0.05. **b** Venn diagram of overlapping DE junctions between each pairwise comparison. **c** Diagram of *Hapln2* gene with splice junctions represented by different letters (A, A’, B, B’, C, C’, D, E, F, and F’). **d** RNA-Seq average counts (reads per million) of *Hapln2* junctions between C (*n* = 6) and W (*n* = 6) groups analyzed by Student’s t-test. No multiple testing correction was applied. **e**, **f** qPCR validation of junctions C and C’ for male (*n* = 10/group) and female rats (*n* = 6/group). **g** Identification of an intron retention event in *Hapln2*. The forward primer was located in exon 3 and the reverse primer in exon 4 (blue arrows). PCR was performed using male samples only and products visualized on agarose gel. The magnified portion of the *Hapln2* gene diagram illustrates the location of splice junctions (C and C’) between exons 3 and 4. Agarose gel showing three bands that after sequencing were identified as containing C’ (200 bp), C (300 bp) and intron retention (500 bp). **h** qPCR of intron retention junction for male and female rats. Relative abundance of junction C (**i)**, C’ (**j**) and intron 3 retention (**k**) calculated as percentage of spliced isoform (PSI) in the hippocampus of male rats. **l** Significant positive correlation between *Pcbp1* mRNA levels and PSI of junction C’ in male rats. Data are presented as the mean ± SEM. *p < 0.05, **p < 0.01 and ****p < 0.0001 by Sidak’s test after two-way ANOVA or by Student’s *t* test.

Sample size was estimated by power analysis using G*Power software [32]. Statistical testing was performed using Prism 9 (GraphPad, San Diego, CA, USA). Statistical outliers were determined using the ROUT method [25] and removed prior to performing comparisons as described above. Equal variance among groups was checked by Bartlett’s test and data were analyzed by two-way ANOVA for variables of treatment and sex, followed by Sidak’s post hoc multiple comparisons test if there was a significant interaction. If sex differences were not found, data was collapsed by sex and analyzed by Student’s *t* test. A *p* value < 0.05 was considered statistically significant. Results of two-way ANOVAs for heatmaps in Fig. 1 are provided in Supplementary Table 4. *p* Values were adjusted for multiple comparisons within a gene. Association was tested by using Pearson correlation (Fig. 2l). Rat experiments were repeated 2–3 times and the data are presented as the mean ± SEM from combined experiments.

## Results

### Altered expression of genes encoding splicing factors in the hippocampus of ethanol withdrawn rats after chronic exposure and human subjects with AUD

We recently published results from RNA-Seq of the hippocampus of male rats during withdrawal from chronic ethanol exposure [11]. In the present study, we explore one of the top enriched pathways from this published data that was not previously investigated, the “spliceosome”. The spliceosome is a large ribonucleoprotein complex that detects splicing signals and catalyzes the removal of non-coding intronic sequences [33]. Differentially expressed genes from the RNA-Seq data in this pathway included *Pcbp1, Snrpa, Snrpb, Eif4a3, Alyref, Lsm4, Sf3a2, Ptbp1*, and *Pcbp2*, which were all increased in the hippocampus during withdrawal from chronic ethanol exposure and included core spliceosome components as well as auxiliary splicing factors. To validate the RNA-Seq data, we performed qPCR to measure expression of these genes in the hippocampus of both male and female rats from the three treatment groups (control, chronic ethanol, and withdrawal). We found increased expression of *Pcbp1, Snrpa, Alyref, and Sf3a2* in males during withdrawal from chronic ethanol exposure compared to male controls (Fig. 1a and Supplementary Fig. 1). In contrast, in females, *Pcbp1, Snrpa, Alyref*, and *Sf3a2* were significantly decreased during withdrawal compared to controls (Fig. 1a, c, Supplementary Fig. 1). Additionally, *Snrpb, Eif4a3, Ptbp1* and *Pcbp2* were not changed in males during withdrawal but were downregulated in females during withdrawal when compared to controls (Fig. 1a, c, Supplementary Fig. 1). Complete results from statistical analyses are shown in Supplementary Table 4. These results suggest a sexually dimorphic effect of ethanol withdrawal on RNA splicing.

To investigate whether the splicing machinery is also altered in humans diagnosed with AUD, we performed qPCR to measure the expression of genes encoding splicing factors in postmortem hippocampus from control (males = 17, females = 7) and AUD (male = 15, females = 10) subjects (Fig. 1b and Supplementary Fig. 2). Two-way ANOVA results are presented in Supplementary Table 5. A significant effect of sex (*F* (1, 45) = 5.27, *p* = 0.0263 was found for *SNRPA, SNRPB* (*F* (1, 45) = 6.77, *p* = 0.0125) and *LSM4* (*F* (1, 45) = 4.33, *p* = 0.0431), but no effect of AUD diagnosis or a sex by diagnosis interaction. For *PCBP1*, there was a significant effect of diagnosis (*F* (1, 43) = 4.49, *p* = 0.0398), however there was no effect of sex or an interaction. *PCBP1* expression was significantly elevated in the hippocampus of individuals with AUD compared to controls (Fig. 1d). No significant effects of sex, treatment and interaction were observed for *EIF4A3*, *SF3A2*, *PCBP2*, and *PTBP1*.

### Differential junction expression in the hippocampus of chronic ethanol exposed and withdrawn rats

We hypothesized that changes in splicing factors during withdrawal from chronic alcohol exposure would result in altered RNA splicing, so we analyzed DE junctions from the male rat hippocampus RNA-Seq data in control, chronic ethanol, and withdrawal groups. Lists of DE junctions are in Supplementary File 1. Out of 148,636 mapped junctions, a total of 1044 DE junctions with FDR < 0.05 were identified (Table 2 and Fig. 2a). 76% of the DE junctions (790) were found between ethanol vs control comparison, while only 10% (108) were DE between the withdrawal vs control comparison. The remaining 14% (146), were DE between the withdrawal vs ethanol comparison (Fig. 2b). Only 34 of the DE junctions were shared between the ethanol vs. control and withdrawal vs. control comparison. These results indicate that chronic ethanol exposure results in a higher number of DE junctions than withdrawal from chronic ethanol exposure and that the majority of these DE junctions are not shared between these conditions.

**Table 2:** Summary of total and differentially expressed junctions (DEJ) identified from RNA-Seq of dorsal hippocampus male rats fed with control liquid diet, ethanol liquid diet, or ethanol liquid diet followed by 24 h of withdrawal from ethanol diet.

Comparisons	Total Junctions	DEJ (*q* < 0.05)	DEJ Upregulated	DEJ Downregulated	Unique Genes
Ethanol vs Control		790	630	160	405
Withdrawal vs Control		108	81	27	53
Ethanol vs Withdrawal		146	59	87	105
TOTAL	148,636	1044	-	-	-

The 108 DE junctions that were found in the withdrawal vs. control comparison mapped to 53 unique annotated genes. We performed pathway analysis on these 53 genes using Enrichr [34] (Supplementary File 2). The top two enriched Reactome pathways were signal transduction and axonal growth inhibition. Genes enriched in these pathways control different steps of peripheral myelination and axonal growth (e.g., *Lingo1, Pde4a, Mag, Cyld, Lama4*) [35].

Among those 53 genes with DE junctions, *Hapln2* (hyaluronan and proteoglycan link protein 2) had 10 possible junctions identified by STAR alignment of the RNA-Seq data (Fig. 2C). Junctions A (*t* = 2.68, df = 10, *p* = 0.022), B (*t* = 3.13, df = 10, *p* = 0.010), B’ (*t* = 3.94, df = 10, *p* = 0.002), C (*t* = 4.14, df = 10, *p* = 0.002), D (*t* = 4.28, df = 10, *p* = 0.001), E (*t* = 3.83, df = 10, *p* = 0.003), and F (*t* = 2.68, df = 10, *p* = 0.022) had the highest read counts per million and increased counts in the withdrawal compared to control group (Fig. 2d). qPCR with primers spanning each of the junctions in a larger group of rats (*n* = 10) demonstrated that junctions A, A’, B, B’, C, and C’ were increased during withdrawal, whereas junctions D, E, F, and F’ were not changed between withdrawal and control conditions (Supplementary Fig. 3). These results indicate increased expression of specific isoforms of *Hapln2* during withdrawal after chronic ethanol exposure in the hippocampus of male rats.

The protein coding sequence for HAPLN2 initiates at exon 3, in which changes in splicing can potentially affect protein sequence. Taking this into consideration, we focused our analysis on junction C, the annotated junction joining exons 3 and 4 in the NCBI reference sequence for *Hapln2*, and junction C’, a previously unannotated junction which results from the use of an alternative 3’ splice site located in exon 4. We performed qPCR with a common forward primer in exon 3 and two reverse primers spanning either junction C or C’ (Supplementary Table 3), on hippocampal RNA from male and female rats. Two-way ANOVA showed a significant sex by treatment interaction (*F* (1, 27) = 6.45, *p* = 0.017), for junction C, with females in withdrawal presenting decreased levels compared to males in withdrawal (*p* = 0.029). For junction C’, there were main effects of sex (*F* (1, 28) = 25.64, *p* < 0.0001), treatment (*F* (1, 28) = 5.25, *p* = 0.0296) and an interaction (*F* (1, 28) = 34.63, *p* < 0.0001). Male rats in withdrawal had higher levels of C’ compared to control males (*p* = 0.039), whereas female rats in ethanol withdrawal had lower levels of C’ compared to control females (*p* = 0.0001) (Fig. 2f).

We then amplified transcripts using PCR in the male rat hippocampal samples using a forward primer located in exon 3 and a reverse primer located in exon 4 to amplify all isoforms and sequenced the resulting PCR products to confirm the presence of the C and C’ junctions. PCR products were separated on an agarose gel and visualized prior to excising the bands and performing Sanger sequencing. We observed three bands on the gel with approximate molecular weights of 200, 300, and 500 bp (Fig. 2g). The 200 and 300 bp bands were transcripts containing the C’ and C junctions, respectively, as predicted. Interestingly, the 500 bp PCR product contained intronic sequence, indicating an additional intron retention variant. This was not due to genomic DNA contamination, as RNA samples were treated with DNase prior to reverse transcription. To quantify this intron retention event between treatment conditions, we performed qPCR in hippocampal RNA samples from male and female control and ethanol-withdrawn rats using a forward primer located in the intron and a reverse primer located in exon 4. There was a significant effect of sex and a sex by treatment interaction (sex, *F* (1, 28) = 23.26, *p* < 0.0001; interaction, *F* (1, 28) = 23.60, *p* < 0.0001). Within males, ethanol withdrawn rats had increased intron retention when compared to controls (*p* = 0.001), whereas within females, ethanol withdrawn rats had lower intron retention compared to controls (*p* = 0.038). Male ethanol withdrawn rats also had significantly greater intron inclusion than female ethanol withdrawn rats (*p* < 0.0001) (Fig. 2h).

Finally, we quantified the relative abundance of each of the PCR products that were amplified in the male rat hippocampus with the universal exon 3 and 4 primers on the agarose gel as the PSI. We found that the isoforms that contained either the canonical junction C or the novel junction C’ were present at higher levels during alcohol withdrawal compared to control (C, *t* = 2.535, df = 16, *p* = 0.0221; C’, *t* = 4.217, df=16, *p* < 0.001). In contrast, the isoform containing the intron was decreased during alcohol withdrawal compared to control (Fig. 2i–k; *t* = 3.900, df = 16, *p* = 0.0013). We performed a Pearson correlation to determine if isoform abundance was associated with levels of *Pcbp1* mRNA in male rats (Fig. 2l). There was a significant positive correlation between *Pcbp1* mRNA levels and abundance of the junction C’ isoform in the withdrawal group (*r* = 0.62, *p* = 0.02) but not in the control group (*r* = 0.04, *p* = 0.55). Correlations were not observed between *Pcbp1* expression and the isoforms containing either intron 3 (*r* = 0.16, *p* = 0.092) or the canonical junction C (*r* = 0.05, *p* = 0.34). Together, these results suggest that during withdrawal after chronic ethanol exposure, alternative splicing of the *Hapln2* transcript is increased in the hippocampus of male rats and decreased in females.

### *HAPLN2* splicing is altered in postmortem hippocampus of human subjects with AUD

**Fig. 3:**
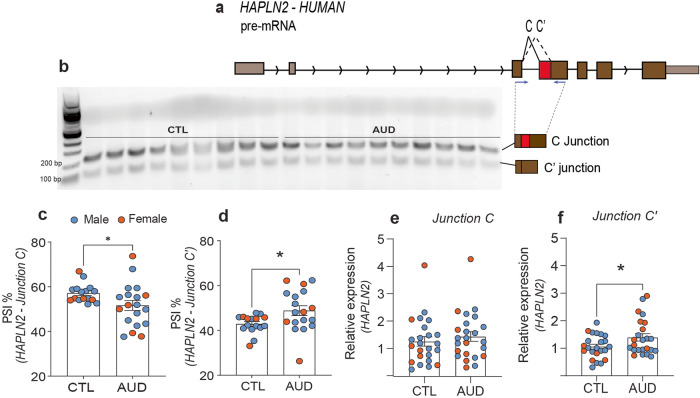
*HAPLN2* splicing is altered in postmortem hippocampus of human subjects with Alcohol Use Disorder (AUD). Human postmortem hippocampus of 24 control (*n* = 17 males and 7 females) and 25 AUD (*n* = 16 males and 9 females) was used for qPCR of *HAPLN2* junctions. **a** Diagram of *HAPLN2* gene depicting junctions C and C’. Primers were located in exons 3 and 4 and are indicated by blue arrows. **b** Agarose gel shows two bands that after sequencing were identified as containing junctions C’ (150 bp) and C (250 bp). Relative abundance of junctions C (**c**) and C’ (**d**), calculated as percentage of spliced isoform (PSI). Relative expression by qPCR with primers spanning junctions C (**e**) and C’ (**f**). Data are presented as the mean ± SEM. *p < 0.05 by Student’s t-test.

To evaluate whether *HAPLN2* in humans undergoes similar differential splicing due to chronic alcohol drinking, we first compared the *HAPLN2* sequence from human and rat. The sequences and splice sites were conserved. In addition, the NCBI database shows a predicted *HAPLN2* transcript variant containing the same alternative C’ splice site as we discovered in the rat (XM_047427123.1; Fig. 3a). We amplified transcripts using primers located in exons 3 and 4 from the postmortem hippocampus of control (male = 17, female = 7) and AUD (male = 16, female = 9) subjects and analyzed them on an agarose gel. As expected, we amplified ~150 bp and ~250 bp fragments that are predicted to contain the C’ and C junctions, respectively (Fig. 3b). We quantified the relative abundance of each PCR product on the gel as PSI. As no sex difference was observed by two-way ANOVA (sex effect, *F* (1, 32) = 0.003, *p* = 0.950), data was collapsed by sex for statistical analysis. We found that the relative abundance of the isoform containing junction C’ was increased in the hippocampus of AUD vs control subjects (*t* = 2.361, df = 32, *p* = 0.0245), while the relative abundance of the isoform containing junction C was correspondingly decreased in AUD vs control (*t* = 2.361, df = 32, *p* = 0.0245).

To further analyze the junction C and C’ levels between control and AUD hippocampus, we performed qPCR using unique primers spanning either junction C or C’. We did not find any sex differences by two-way ANOVA (junction C: *F* (1, 44) = 0.08, *p* = 0.7698; junction C’: *F* (1, 44) = 0.14, *p* = 0.7024), so we collapsed the data by sex for statistical analysis. The levels of junction C did not differ between the AUD and control groups (*t* = 0.7203, df = 46, *p* = 0.4750) (Fig. 3e). However, junction C’ was more abundant in the hippocampus of AUD compared to control subjects (*t* = 2.075, df = 45, *p* = 0.0437) (Fig. 3f). All together, these results indicate that splicing occurs more frequently at the *HAPLN2* C’ site in humans diagnosed with AUD and suggests that this might result in increased translation of a non-functional, truncated protein product.

### PCBP1 binding is enriched at *HAPLN2* splice sites during withdrawal from chronic ethanol exposure in the hippocampus of male rats and AUD subjects

**Fig. 4:**
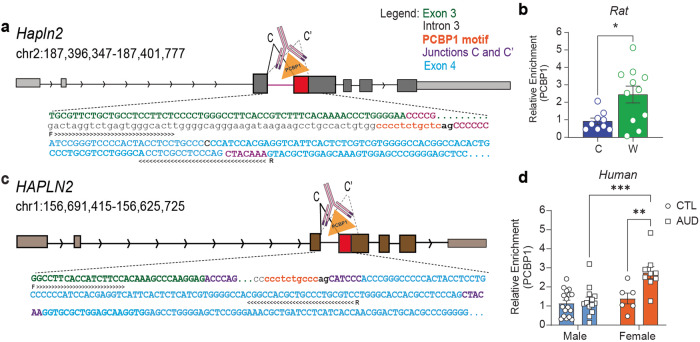
PCBP1 binding is enriched at *HAPLN2* splice sites during withdrawal from chronic ethanol exposure in the hippocampus of male rats and AUD subjects. Male rats were fed control liquid diet (C, *n* = 9), or ethanol liquid diet followed by 24 h of withdrawal from ethanol diet (W, *n* = 11). The dorsal hippocampus was dissected and subjected to RNA immunoprecipitation (RIP) with a PCBP1 antibody. **a** Rat *Hapln2* gene structure and illustration of the RIP method. **b** Graph of the relative enrichment of PCBP1 association near the C and C’ splice junctions in male rat *Hapln2*. *p < 0.05 by Student’s *t* test. **c** Human *HAPLN2* gene structure and illustration of the RIP method. **d** Graph of the relative enrichment of PCBP1 around C and C’ junctions in human *HAPLN2*. Postmortem human hippocampus tissue from control (CTL, *n* = 23, 6 females and 17 males) and AUD (*n* = 25, 10 females and 15 males) were used for RIP with a PCBP1 antibody. Two samples were detected as outliers in the male AUD group and one in the female AUD group and were excluded. **p < 0.001 and ****p < 0.0001 by Sidak’s test after two-way ANOVA. Data are presented as the mean ± SEM.

Given that *PCBP1* expression increased in the hippocampus of male rats during withdrawal from chronic ethanol exposure and human subjects with AUD, and that *HAPLN2* splicing was similarly altered in these conditions, we hypothesized that PCBP1 might be involved in alternative splicing of *HAPLN2*. PCBP1 regulates RNA splicing by binding to cytosine-rich regions surrounding splice sites [12, 36]. A PCBP1 binding motif was found adjacent to the 3’ splice site for junction C in both rat and human *HAPLN2* (Fig. 4a, c). To determine if PCBP1 associates with the rat *Hapln2* pre-mRNA, RIP was performed in the hippocampus of both male and female rats, using a PCBP1 antibody [29, 30] followed by qPCR using primers surrounding the cytosine rich PCBP1 motif in *Hapln2* (Fig. 4a). Interestingly, PCBP1 binding was enriched in this region during withdrawal in male rats (*t* = 3.526, df = 8, *p* = 0.0078), implicating it in the regulation of *Hapln2* splice junction usage (Fig. 4b). No qPCR signal was detected for *Hapln2* after PCBP1 RIP in the hippocampus of female rats, although this is not surprising considering that *Pcbp1* expression was significantly decreased during withdrawal in females.

We next performed RIP using the PCBP1 antibody in postmortem human hippocampal AUD and control samples. We observed a significant effect of sex (*F* (1, 41) = 12.77, *p* = 0.0009), diagnosis (*F* (1, 41) = 8.99, *p* = 0.0046) and an interaction (*F* (1, 41) = 6.41, *p* = 0.0152), with females diagnosed with AUD presenting increased enrichment of PCBP1 binding around the *HAPLN2* exon 4 splice sites when compared with control females (*p* = 0.003) and males diagnosed with AUD (*p* < 0.0001) (Fig. 4d). Together, these results provide evidence that PCBP1 regulates *HAPLN2* alternative splicing in the hippocampus in rats and humans.

## Discussion

In this study we provide evidence for a conserved molecular mechanism for altered RNA splicing following chronic ethanol exposure in the hippocampus of rats and humans. *PCBP1* expression was increased in the hippocampus during withdrawal from chronic ethanol exposure in male rats and in postmortem hippocampus of AUD subjects. Similarly, *HAPLN2* was alternatively spliced at a site in exon 4 associated with PCBP1 binding in both species. These results indicate a similarity between mechanisms in rats and humans that drive altered RNA processing in the hippocampus in response to chronic ethanol exposure.

Of note, there were sex differences in the expression of splicing factors and alternative splicing in the hippocampus of rats during withdrawal from chronic ethanol exposure. Expression of genes encoding splicing factors were generally increased in males and decreased in females during withdrawal. Likewise, usage of the *Hapln2* C’ splice site was increased in male rats and decreased in female rats during withdrawal, and PCBP1 association with *Hapln2* pre-mRNA was only observed in male rats. Similarly, a recent study reported an enrichment of DE genes in the spliceosome pathway in male, but not female mice treated with a single dose of ethanol [37]. The biological factors that underly sex differences in RNA processing are not known, but estrogen plays an important role in the rodent hippocampus in molecular and cellular mechanisms driving learning and memory. Therefore, is possible that hormonal factors are involved [38], although genetic factors could also be relevant.

We did not observe the same sex differences in the expression of splicing factors and *HAPLN2* alternative splicing in human hippocampus. In contrast to rats, *PCBP1* expression and *HAPLN2* alternative splicing were elevated in the hippocampus of both sexes diagnosed with AUD. With respect to potential effects of hormones on RNA processing, the lack of a sex difference in these measures in human samples could be due to the age of women at the time of death. The average age was 58 (AUD)–59 (control), with a range from 43 to 78. As a result, the women may have been either in the perimenopausal transition or post-menopausal and any effects of ovarian hormones may have been absent. The differences in our results between humans and rats could be due to several factors, such as the difference in the pattern and timing of ethanol exposure between rats and humans. We also found a greater association of PCBP1 with the *HAPLN2* splice site in the postmortem hippocampus of women with AUD. Sex differences in alternative splicing in human brain have been described, highlighting the importance of analyzing samples from both sexes [39, 40].

In the male rat hippocampus, it appeared as if the expression of *Hapln2* at the 5’ end of the gene was increased during withdrawal. Transcription and alternative splicing are known to be intimately coupled [41]. Splicing can be controlled by the rate of RNA polymerase processivity and epigenetic factors [42]. We previously published that histone deacetylase 2 (HDAC2) levels are increased in the rat hippocampus during withdrawal from chronic ethanol exposure [43], so it is possible, for example, that increased transcription and alternative splicing of *Hapln2* are related to changes in HDAC2 or other epigenetic enzymes during withdrawal. The other possibility is that alternative splicing of *Hapln2* could be increasing its transcription, as there are examples in the literature of this phenomenon [41]. Finally, PCBP1 is also known to affect gene expression in addition to alternative splicing [14].

HAPLN2 (also known as Bral1) is an extracellular matrix protein that links chondroitin sulfate proteoglycans to hyaluronan and is primarily expressed by oligodendrocytes [44]. It is located at nodes of Ranvier in myelinated white matter and maintains the extracellular diffusion barrier [45]. Neuronal conductivity is reduced in *Hapln2* deficient mice [45], demonstrating an important role for this protein in neurotransmission. Increased use of the C’ splice site and intron retention in *Hapln2* during withdrawal from chronic ethanol exposure is predicted to result in a frameshift, stop codon introduction, and production of truncated protein products (likely resulting in a loss of function). In the rat RNA-Seq data, we also found DE junctions in the *Mag* and *Lingo1* genes, which encode myelin sheath proteins, during withdrawal from chronic alcohol exposure (Supplementary file 2). As chronic ethanol exposure leads to changes in myelination that are accompanied by deficits in cognition and emotion regulation [45–48], these results suggest that alternative splicing of myelin-associated and ECM proteins could contribute to cognitive and emotional deficits during withdrawal from chronic ethanol exposure. The behavioral and cellular consequences of alternative *Hapln2* splicing during withdrawal will be explored in future studies in rats.

We found that withdrawal from chronic ethanol exposure led to a marked increase in *Pcbp1* mRNA levels in the hippocampus of male rats. Higher *PCBP1* expression was also seen in the postmortem hippocampus of AUD subjects of both sexes. A causal role for PCBP1 in alcohol drinking and withdrawal-associated behaviors needs to be investigated in future studies in rats. PCBP1 binding was enriched around the *HAPLN2* exon 4 splice sites in ethanol-exposed male rat and in the postmortem hippocampus of women diagnosed with AUD. We hypothesize that during ethanol withdrawal, PCBP1 binds to a cytosine-rich sequence at the C junction splice site and inhibits its usage, resulting in increased usage of the C’ site. Supporting this hypothesis, Wang and collaborators [49] showed that overexpression of PCBP1 changes splice site usage at *STAT3* exon 23 by binding to an exonic splicing suppressor site, promoting the switch from the oncogenic longer *STAT3α* isoform to the shorter tumor-suppressive *STAT3β* isoform. With regard to a potential PCBP1-*STAT3* interaction, increased expression of *STAT3* in the hippocampus of ethanol-withdrawn rats was also observed in our RNA-seq data, as well as in postmortem hippocampus of human AUD subjects [11]. It is possible that PCBP1 could regulate *STAT3* expression and/or splicing during alcohol withdrawal in rats and in AUD subjects, although this requires further investigation.

PCBP1 is expressed ubiquitously in cells throughout the brain, including in oligodendrocytes. In addition to regulating RNA splicing, PCBP1 regulates trafficking and translation of RNAs in oligodendrocytes [50]. Here we have provided evidence that in addition to the known role of PCBP1 in suppressing RNA translation, PCBP1 likely regulates alternative splicing of oligodendrocyte-expressed genes. Studies in PC12 cells have also demonstrated that PCBP1 regulates the expression and RNA splicing of genes involved in neuroinflammation [14]. It is well documented that chronic ethanol exposure induces a brain immune response [51, 52], thus PCBP1 could also function in other brain cell types to regulate the expression and splicing of immune-response genes. More investigation is needed to determine the different functions of PCBP1 in ethanol-responsive RNA processing and to identify additional genome-wide targets of PCBP1 in the nervous system during withdrawal from chronic ethanol exposure. Finally, it will be important to determine if PCBP1 in oligodendrocytes or other brain cell types is involved in altered cognitive and emotional behaviors observed during withdrawal from chronic ethanol exposure. This study demonstrates that PCBP1 plays an important role in aberrant RNA splicing in the brain of rats and humans after chronic ethanol exposure and suggests that alternative splicing of myelin-associated genes could be associated with white matter deficits induced by chronic alcohol exposure.

### Supplementary information

Supplementary figure 1

Supplementary figure 2

Supplementary figure 3

Supplementary file 1

Supplementary file 2

Supplementary information
