# Supplementary figures and images for: Conserved role for PCBP1 in altered RNA splicing in the hippocampus after chronic alcohol exposure
Source: Mol Psychiatry. 2023 Aug 3;28(10):4215–24. doi: 10.1038/s41380-023-02184-y (PMC10827656; doi:10.1038/s41380-023-02184-y)

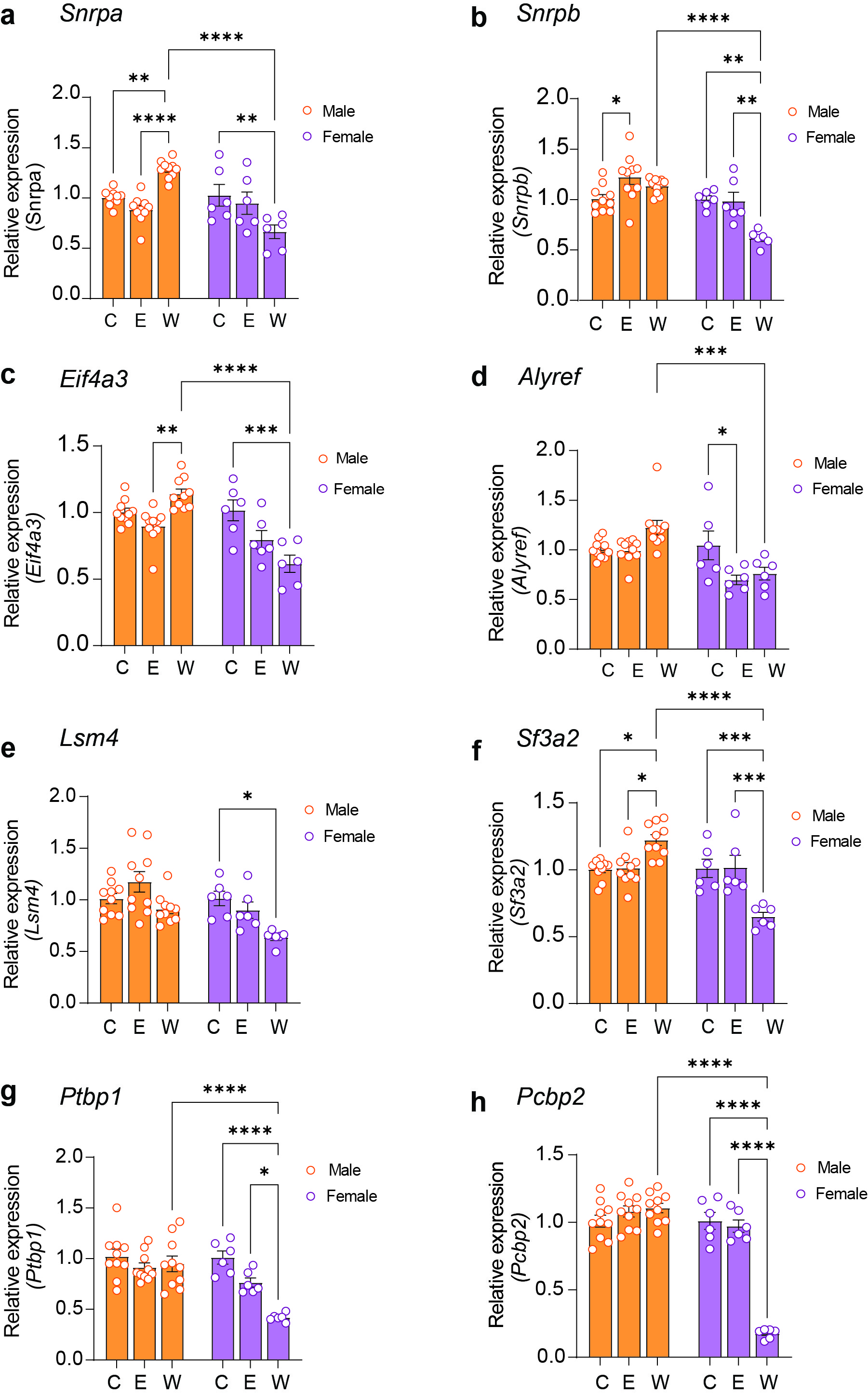

Supplement: Supplementary file 1 — Supplementary figure 1 [file 41380_2023_2184_MOESM1_ESM.jpg]

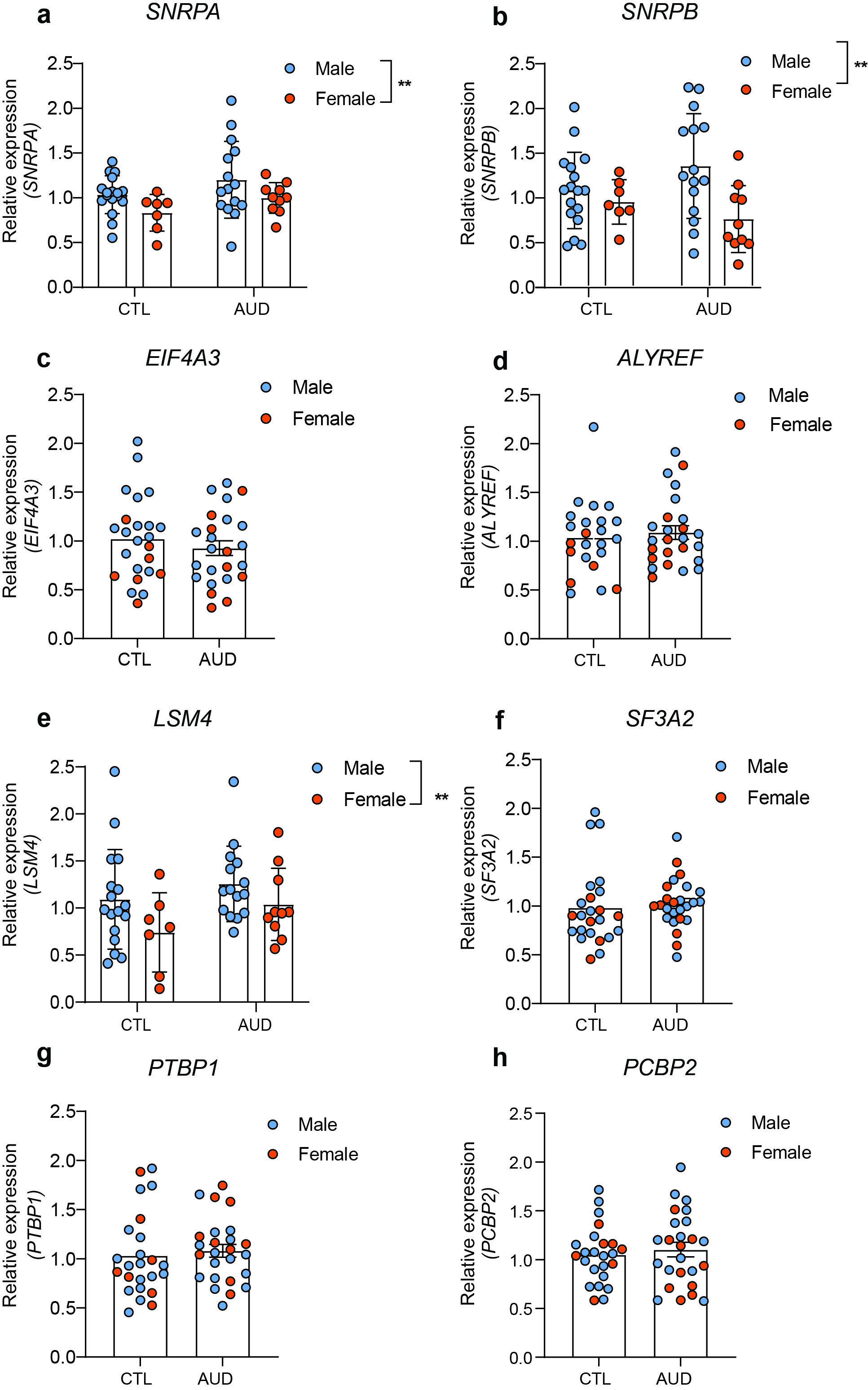

Supplement: Supplementary file 2 — Supplementary figure 2 [file 41380_2023_2184_MOESM2_ESM.jpg]

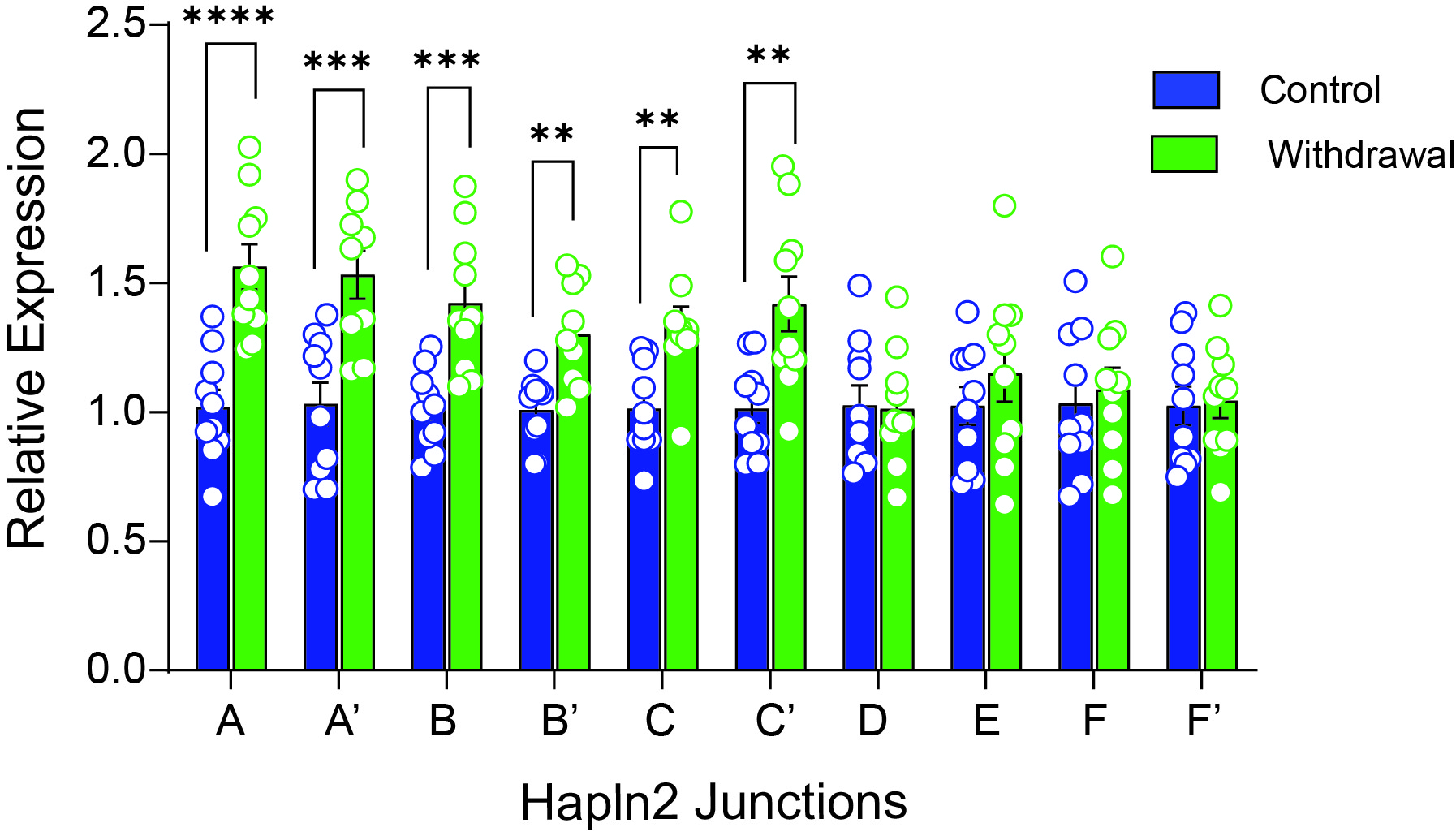

Supplement: Supplementary file 3 — Supplementary figure 3 [file 41380_2023_2184_MOESM3_ESM.jpg]
