# Supplementary material for: Conserved role for PCBP1 in altered RNA splicing in the hippocampus after chronic alcohol exposure
Source: Mol Psychiatry. 2023 Aug 3;28(10):4215–24. doi: 10.1038/s41380-023-02184-y (PMC10827656; doi:10.1038/s41380-023-02184-y)
Supplement: Supplementary file 6 — Supplementary information [file 41380_2023_2184_MOESM6_ESM.docx]

**Supplementary Information**

**Conserved role for PCBP1 in altered RNA splicing in the hippocampus after chronic alcohol exposure.**

**Running title:** Ethanol withdrawal and alternative splicing

Luana Martins de Carvalho^1*#^, Hu Chen^1^, Mark Maienschein-Cline^1,3^, Elizabeth J. Glover^1^, Subhash C. Pandey^1,2^, and Amy W. Lasek^1#^

^1^Center for Alcohol Research in Epigenetics, Department of Psychiatry, University of Illinois at Chicago, Chicago, IL 60612

^2^Jesse Brown VA Medical Center, Chicago, IL 60612

^3^Research Informatics Core, University of Illinois at Chicago, Chicago, IL 60612

*Correspondence should be addressed to Luana Carvalho at [luana.martinsdecarvalho@vcuhealth.org](mailto:luana.martinsdecarvalho@vcuhealth.org)

**Table S1. Primer sequences for rat genes.**

| **Gene** | **Forward Primer (5’ – 3’)** | **Reverse Primer (5’ – 3’)** |
| --- | --- | --- |
| *Pcbp1* | TTTGCCGGATTCTCAGGGGT | CGCTTTCAACCCCGCGT |
| *Snrpa* | CCCGTCCCAACCACACTATT | GCACTGCTGACCTCCTTGAA |
| *Snrpb* | TGCAGCACATCGACTACAGG | TCCTCGAAGCAACACCAGAC |
| *Eif4a3* | GCACACCAGGACGAGTCTTT | GCGCTGATGAGAACAACCTG |
| *Alyref* | TGTTTGCCGAGTTTGGAACG | AGCTGGATGTTCATAGGGCG |
| *Lsm4* | CGCCTTATCCCGTTCCGATTG | TCTTCAGCAGCGACAAGGGA |
| *Sf3a2* | CGGCATTCGGGGAGGTTAAG | GATGCAGGCCTGGGTACTTT |
| *Ptbp1* | AGCCTTTGGCCTCTCTGTTC | TGCTGACCAAAAGGACGGAA |
| *Pcbp2* | GCCTATTGGGCAGGTTTGGA | CTGACGCCCAATTATGCAGC |
| *Hprt* | TCCTCAGACCGCTTTTCCCGC | TCATCATCACTAATCAGACGCTGG |
| *Gusb* | TCCTTCCATGTATCCCAAGG | TGGTAGGGGTGGTGTACAGG |

**Table S2. Primer sequencing for human genes.**

| **Gene** | **Forward Primer (5’ – 3’)** | **Reverse Primer (5’ – 3’)** |
| --- | --- | --- |
| *PCBP1* | TCCATGACCAACAGTACCGC | TACACCCGCCTTTCCCAATC |
| *SNRPA* | GTCCCTGTACGCCATCTTCTC | GTCGGTCTTGGCATACTGGAT |
| *SNRPB* | GCCCACCTCCTGGTATGAGA | GTAACGTGGCCCTGAGGAAT |
| *EIF4A3* | GCAACGAGCAATCAAGCAGAT | CAACTCTCTTGTGGGAGCCAA |
| *ALYREF* | GGGAAACTGCTGGTGTCCAA | GTTCCTAAGCTGCGACCAGA |
| *LSM4* | AGCTGCGACAACTGGATGAA | AGGTACTTGATGGTGCTGCC |
| *SF3A2* | AGAACCACCTGGGCTCCTAT | TGCTTCTTCCCCTGCGTATG |
| *PTBP1* | GGGTCTGCTCTGTGTGCC | ACTTCGGCTGTCACCTTTGA |
| *PCBP2* | ATCTGCGTGGTCATGTTGG | GCCATGCGTCATGGGAAAAT |
| *B2M* | CTATCCAGCGTACTCCAAA | GCTCCACTTTTTCAATTCTC |
| *ACT* | CTCCCTGGAGAAGAGCTAC | GATCCACACGGAGTACTTG |

**Table S3. Primer sequences for *Hapln2* junctions.**

| ***Hapln2* Junctions - Rat** | **Forward Primer (5’ – 3’)** | **Reverse Primer (5’ – 3’)** |
| --- | --- | --- |
| C | GGGCCTTCACCGTCTTTCA | GGATGGGGGGCGGGG |
| C’ |  | CAGCGTACTTTGTAGCGGGG |
| Intron 3 retention/ PSI | GGGCCTTCACCAGCTTTCACA | ATGATCAGGGAGGCGTCCAGC |
| ***HAPLN2* Junctions - Human** | **Forward Primer (5’ – 3’)** | **Reverse Primer (5’ – 3’)** |
| C | GGGCCTTCACCATCTTCCACAAA | CGGGTGGGATGCTGGGTCT |
| C’/PSI |  | CGCACCTTGTAGCTGGGTCT |

**Table S4. Two-way ANOVA statistics for qPCR data in Fig. 1A.**

| **Gene** | **Gene expression** | | | **Post Hoc p values** | | | | |
| --- | --- | --- | --- | --- | --- | --- | --- | --- |
|  | **Effect of Sex** | **Effect of Treatment** | **Interaction** | **Within Sex** | | | **Between Sex** | |
| *Pcbp1* | F (1, 42) = 25.64, p<0.0001 | F (2, 42) = 1.07,  p=0.3499 | F (2, 42) = 16.99,  p<0.0001 | **M** C vs. E | >0.9999 | | C **M vs. F** | 0.9895 |
|  |  |  |  | C vs. W | 0.0033 | |  |  |
|  |  |  |  | E vs. W | 0.0031 | | E **M vs. F** | 0.3793 |
|  |  |  |  | **F**  C vs. E | 0.3154 | |  |  |
|  |  |  |  | C vs. W | 0.0003 | | W **M vs. F** | <0.0001 |
|  |  |  |  | E vs. W | 0.0281 | |  |  |
| *Snrpa* | F (1, 42) = 13.02,  p=0.0008 | F (2, 42) = 1.36,  p=0.2665 | F (2, 42) = 20.42,  p<0.0001 | **M** C vs. E | 0.3017 | | C **M vs. F** | 0.9894 |
|  |  |  |  | C vs. W | 0.0012 | |  |  |
|  |  |  |  | E vs. W | <0.0001 | | E **M vs. F** | 0.8329 |
|  |  |  |  | **F**  C vs. E | 0.8006 | |  |  |
|  |  |  |  | C vs. W | 0.0014 | | W **M vs. F** | <0.0001 |
|  |  |  |  | E vs. W | 0.0142 | |  |  |
| *Snrpb* | F (1, 42) = 30.82,  p<0.0001 | F (2, 42) = 8.43,  p=0.0008 | F (2, 42) = 10.57  p=0.0002 | **M** C vs. E | 0.0090 | | C **M vs. F** | >0.9999 |
|  |  |  |  | C vs. W | 0.1987 | |  |  |
|  |  |  |  | E vs. W | 0.4898 | | E **M vs. F** | 0.0131 |
|  |  |  |  | **F**  C vs. E | 0.9960 | |  |  |
|  |  |  |  | C vs. W | 0.0002 | | W **M vs. F** | <0.0001 |
|  |  |  |  | E vs. W | 0.0004 | |  |  |
| *Eif413* | F (1, 42) = 24.67,  p<0.0001 | F (2, 42) = 5.93,  p=0.0054 | F (2, 42) = 15.74,  p<0.0001 | **M** C vs. E | 0.2528 | | C **M vs. F** | 0.9972 |
|  |  |  |  | C vs. W | 0.0962 | |  |  |
|  |  |  |  | E vs. W | 0.0010 | | E **M vs. F** | 0.4086 |
|  |  |  |  | **F**  C vs. E | 0.0247 | |  |  |
|  |  |  |  | C vs. W | <0.0001 | | W **M vs. F** | <0.0001 |
|  |  |  |  | E vs. W | 0.0847 | |  |  |
| *Alyref* | F (1, 42) = 17.86,  p=0.0001 | F (2, 42) = 3.83,  p=0.0296 | F (2, 42) = 6.87,  p=0.0026 | **M** C vs. E | 0.9988 | | C **M vs. F** | 0.9654 |
|  |  |  |  | C vs. W | 0.0398 | |  |  |
|  |  |  |  | E vs. W | 0.0285 | | E **M vs. F** | 0.0128 |
|  |  |  |  | **F**  C vs. E | 0.0082 | |  |  |
|  |  |  |  | C vs. W | 0.0377 | | W **M vs. F** | <0.0001 |
|  |  |  |  | E vs. W | 0.9168 | |  |  |
| *Lsm4* | F (1, 42) = 9.57,  p=0.0035 | F (2, 42) = 8.15,  p=0.0010 | F (2, 42) = 2.43,  p=0.1003 | N/A | N/A | | N/A | N/A |
| *Sf3a2* | F (1, 42) = 21.39,  p<0.0001 | F (2, 42) = 1.56,  p=0.2216 | F (2, 42) = 22.95,  p<0.0001 | **M** C vs. E | 0.9981 | | C **M vs. F** | 0.9992 |
|  |  |  |  | C vs. W | 0.0023 | |  |  |
|  |  |  |  | E vs. W | 0.0037 | | E **M vs. F** | 0.9998 |
|  |  |  |  | **F**  C vs. E | 0.9998 | |  |  |
|  |  |  |  | C vs. W | 0.0001 | | W **M vs. F** | <0.0001 |
|  |  |  |  | E vs. W | <0.0001 | |  |  |
| *Ptbp1* | F (1, 42) = 18.18,  p=0.0001 | F (2, 42) = 12.74,  p<0.0001 | F (1, 42) = 18.18,  p=0.0009 | **M** C vs. E | | 0.4532 | C **M vs. F** | 0.9992 |
|  |  |  |  | C vs. W | | 0.7492 |  |  |
|  |  |  |  | E vs. W | | 0.9593 | E **M vs. F** | 0.3180 |
|  |  |  |  | **F**  C vs. E | | 0.0665 |  |  |
|  |  |  |  | C vs. W | | <0.0001 | W **M vs. F** | <0.0001 |
|  |  |  |  | E vs. W | | 0.0057 |  |  |
| *Pcbp2* | F (1, 42) = 90.57,  p<0.0001 | F (2, 42) = 47.78,  p<0.0001 | F (2, 42) = 65.22,  p<0.0001 | **M** C vs. E | | 0.4778 | C **M vs. F** | >0.9999 |
|  |  |  |  | C vs. W | | 0.2275 |  |  |
|  |  |  |  | E vs. W | | 0.9560 | E **M vs. F** | 0.2448 |
|  |  |  |  | **F**  C vs. E | | 0.9278 |  |  |
|  |  |  |  | C vs. W | | <0.0001 | W **M vs. F** | <0.0001 |
|  |  |  |  | E vs. W | | <0.0001 |  |  |

N/A. Not applicable as there was no sex by treatment interaction.

**Table S5. Two-way ANOVA statistics for qPCR data in Fig. 1B.**

| **Gene** | **Gene expression** | | |
| --- | --- | --- | --- |
|  | **Effect of Sex** | **Effect of Treatment** | **Interaction** |
| *PCBP1* | F (1, 43) = 0.04, p=0.8408 | F (1, 43) = 4.49, p=0.0398 | F (1, 43) = 0.08, p=0.7723 |
| *SNRPA* | F (1, 45) = 5.27, p=0.0263 | F (1, 45) = 3.51, p=0.0673 | F (1, 45) = 7.793^e-005^, p=0.9930 |
| *SNRPB* | F (1, 45) = 6.77, p=0.0125 | F (1, 45) = 0.08, p=0.7682 | F (1, 45) = 2.81, p=0.099 |
| *EIF413* | F (1, 45) = 0.05, p=0.8113 | F (1, 45) = 0.55, p=0.4600 | F (1, 45) = 1.21, p=0.2771 |
| *ALYREF* | F (1, 45) = 0.0019, p=0.9647 | F (1, 45) = 0.17, p=0.6775 | F (1, 45) = 0.83, p=0.3665 |
| *LSM4* | F (1, 45) = 4.33, p=0.0431 | F (1, 45) = 2.85, p=0.0980 | F (1, 45) = 0.24, p=0.6257 |
| *SF3A2* | F (1, 45) = 1.45, p=0.2208 | F (1, 45) = 0.90, p=0.3460 | F (1, 45) = 1.60, p=0.2021 |
| *PTBP1* | F (1, 45) = 0.03, p=0.8577 | F (1, 45) = 0.84, p=0.3632 | F (1, 45) = 2.61, p=0.1131 |
| *PCBP2* | F (1, 45) = 1.03, p=0.3157 | F (1, 45) = 0.03, p=0.8560 | F (1, 45) = 2.41, p=0.1500 |

**Supplementary figure and file legends**

**Supplementary figure 1.** **Altered expression of genes encoding splicing factors in the hippocampus of ethanol withdrawn rats after chronic exposure. (a-h)** Scatter plot showing the relative expression of *Alyref*, *Ptbp1*, *Snrpa*, *Snrpb*, *Sf3a2*, *Pcbp2*, *Eif4a3* and *Lsm4* relative to the control group in male and female rat hippocampus. Data shown as the mean ± SEM with individual data points as circles. *p < 0.05 and ****p < 0.0001 by Sidak’s test after two-way ANOVA. Full statistics are reported in Supplementary Table 3.

**Supplementary figure 2. Altered expression of genes encoding splicing factors in the hippocampus of human subjects with AUD. (a-h)** Scatter plot showing the relative expression of *PCBP2*, *PTBP1*, *SNRPA*, *SNRPB*, *ALYREF*, *EIF4A3*, *SF3A2*, and *LSM4*. Data shown as the mean ± SEM with individual data points as circles **(d-h)** ** p< 0.005 for significant effect of sex after two-way ANOVA. **(a-c; e-g)** Student’s t-test was used in the absence of sex effect.

**Supplementary figure 3.** qPCR of *Hapln2* junctions between C (n=10) and W (n=10) groups analyzed by Student’s t-test. No multiple testing correction was applied.

**Supplementary file 1**. Lists of differentially expressed junctions.

**Supplementary file 2**. Pathway analysis of the 53 genes containing 108 differentially expressed junctions found in the withdrawal vs. control comparison.
